# Associations of Dietary Patterns and Physical Activity with Sleep Quality and Metabolic Health Markers in Patients with Obstructive Sleep Apnea: An Exploratory Pilot Study

**DOI:** 10.3390/nu18030409

**Published:** 2026-01-26

**Authors:** Li-Ang Lee, Yi-Ping Chao, Ruei-Shan Hu, Wan-Ni Lin, Hsueh-Yu Li, Li-Pang Chuang, Hai-Hua Chuang

**Affiliations:** 1 Department of Otorhinolaryngology-Head and Neck Surgery, Linkou Main Branch, Chang Gung Memorial Hospital, Taoyuan City 33305, Taiwan; 5738@cgmh.org.tw (L.-A.L.); yiping@mail.cgu.edu.tw (Y.-P.C.); sandyhutfg111@gmail.com (R.-S.H.); y1829@cgmh.org.tw (W.-N.L.); hyli38@cgmh.org.tw (H.-Y.L.); 2 School of Medicine, College of Medicine, Chang Gung University, Taoyuan City 33302, Taiwan; lpchuang1678@yahoo.com.tw; 3 School of Medicine, College of Life Science and Medicine, National Tsing Hua University, Hsinchu City 300044, Taiwan; 4 Department of Computer Science and Information Engineering, College of Engineering, Chang Gung University, Taoyuan City 33302, Taiwan; 5 Department of Pulmonary and Critical Care Medicine, Linkou Main Branch, Chang Gung Memorial Hospital, Taoyuan City 33305, Taiwan; 6 Department of Community Medicine, Cathay General Hospital, Taipei City 106438, Taiwan; 7 Department of Industrial Engineering and Management, National Taipei University of Technology, Taipei City 10608, Taiwan

**Keywords:** body composition, cardiometabolic health, chewing frequency, dietary behavior, insulin resistance, metabolic syndrome, obstructive sleep apnea, physical activity, sleep quality, walking

## Abstract

**Background/Objectives:** Obstructive sleep apnea (OSA) is often accompanied by metabolic syndrome (MetS), forming a high-risk phenotype with elevated cardiometabolic burden. The contribution of lifestyle behaviors—particularly eating mechanics and psychological eating cues—to disease severity remains unclear. This study examined independent associations of dietary behaviors and physical activity (PA) with OSA severity, sleep quality, and metabolic health. **Methods:** Forty-four OSA patients (mean age 38.3 ± 9.1 years; 89% male) underwent attended polysomnography, dual-energy X-ray absorptiometry, and metabolic profiling. Validated questionnaires assessed dietary behaviors, PA, and sleep quality. Hierarchical logistic regression identified predictors of MetS, severe OSA, and poor sleep quality. **Results:** The prevalence of MetS was 45%. Compared with those with OSA alone, participants with MetS demonstrated significantly greater central adiposity and more severe nocturnal hypoxemia, despite similar apnea–hypopnea indexes. In multivariable models, MetS was independently associated with higher body mass index (adjusted odds ratio [aOR] = 1.64; *p* = 0.008) and reward eating (aOR = 3.34; *p* = 0.041), whereas higher total PA was associated with reduced odds (aOR = 0.96; *p* = 0.026). Poor subjective sleep quality was significantly associated with younger age (aOR = 0.91; *p* = 0.037). For severe OSA, slow chewing was associated with significantly reduced odds (aOR = 0.24; *p* = 0.038), while emotional eating was associated with increased odds (aOR = 2.40; *p* = 0.048). **Conclusions:** This hypothesis-generating study identifies a high-risk OSA phenotype marked by metabolic dysfunction and hypoxemia. Eating speed (a proxy for mindful eating), emotional and reward-driven eating, and PA independently shape metabolic and respiratory outcomes. These findings support incorporating behavioral nutrition into multidisciplinary OSA management.

## 1. Introduction

Obstructive sleep apnea (OSA) is a prevalent chronic condition characterized by recurrent upper airway collapse during sleep, leading to intermittent hypoxia and sleep fragmentation [1]. It is now recognized not merely as a localized anatomical problem but as a systemic disorder intricately linked to metabolic syndrome (MetS)—a cluster of conditions including central obesity, hypertension, dyslipidemia, and insulin resistance [2]. The pathophysiological connection between OSA and metabolic dysfunction is bidirectional and complex. Chronic intermittent hypoxia triggers sympathetic activation, oxidative stress, and the release of inflammatory cytokines (e.g., tumor necrosis factor-α, interleukin-6), which in turn drive insulin resistance and endothelial dysfunction [3]. Conversely, visceral adiposity exacerbates the disease through two distinct mechanisms: mechanically, by reducing lung volume and upper airway traction; and biologically, by acting as a metabolically active tissue that secretes adipokines, further promoting airway collapsibility [4].

While the association between OSA and metabolic dysfunction is well-established, the specific biological and behavioral mechanisms through which modifiable lifestyle factors—such as dietary patterns and physical activity (PA)—modulate this relationship remain underexplored. Dietary behaviors extend beyond simple caloric intake [5]. Emerging evidence suggests that eating mechanics, such as eating speed and chewing frequency, play a dual role in both metabolic regulation and upper airway function. Metabolically, rapid eating and insufficient chewing may impair cephalic phase insulin release and delay gut hormone signaling (e.g., glucagon-like peptide-1, peptide YY) [6], leading to attenuated satiety and postprandial hyperglycemia [7]. Mechanistically, rigorous mastication recruits the muscles of mastication and the tongue (specifically the genioglossus); thus, we hypothesized that increased chewing frequency might act as a form of passive orofacial myofunctional training, potentially enhancing upper airway muscle tone and reducing collapsibility [8,9].

Similarly, the relationship between PA and OSA severity likely involves mechanisms beyond simple weight management. Sedentary behavior is hypothesized to exacerbate OSA by promoting daytime fluid retention in the legs, which subsequently shifts rostrally to the neck during recumbency, potentially increasing pharyngeal edema and airway resistance [10,11]. Conversely, PA may be associated with improved outcomes through distinct physiological pathways depending on intensity [12]. While moderate-to-vigorous PA is typically linked to improved systemic insulin sensitivity and metabolic fitness [13], low-intensity PA such as walking may specifically help reduce these nocturnal rostral fluid shifts without inducing the exercise intolerance often seen in this population [10]. Given that high-intensity exercise may be poorly tolerated due to OSA-related chronic fatigue [14,15], identifying the benefits of sustainable, low-intensity behaviors like walking is clinically critical.

Despite these mechanistic links [16,17], few studies have comprehensively examined whether specific dietary mechanics (such as chewing frequency) and distinct PA intensities exert differential effects on the respiratory versus metabolic domains of OSA. Most existing research has focused broadly on weight loss or total caloric restriction, often overlooking the independent potential of behavioral eating patterns [11,13]. Furthermore, the specific associations of sedentary time versus walking versus vigorous activity with respiratory parameters (apnea–hypopnea index [AHI]) compared to metabolic parameters (insulin resistance) remain largely uncharacterized in this specific clinical population [18].

Therefore, this hypothesis-generating study aims to investigate the independent associations of dietary patterns and PA with three distinct domains of health in adult patients with OSA: (1) systemic metabolic health (MetS), (2) subjective sleep burden (sleep quality), and (3) respiratory severity (AHI). The rationale for analyzing these as parallel outcomes lies in the clinical heterogeneity of OSA; patients often present with severe obstruction without metabolic comorbidities, or conversely, profound metabolic dysfunction with moderate obstruction. Uniquely, this study integrates behavioral assessments—specifically chewing frequency and walking activity—with high-precision objective measurements, including polysomnography and dual-energy X-ray absorptiometry (DXA). By bridging the gap between daily lifestyle habits and objective physiological outcomes, we aim to explore whether distinct behaviors target different domains of the disease phenotype.

## 2. Materials and Methods

### 2.1. Study Design and Ethical Approval

This study employed a cross-sectional design analyzing data from a prospectively recruited observational cohort. The study protocol was approved by the Institutional Review Board of the Chang Gung Medical Foundation (protocol code 202100455A3C501) and conducted in accordance with the Declaration of Helsinki [19]. Study procedures took place between 1 June 2021, and 31 January 2024. All participants provided written informed consent prior to enrollment. The reporting of this study adheres to the Strengthening the Reporting of Observational Studies in Epidemiology (STROBE) guidelines [20].

### 2.2. Study Participants

**Figure 1:**
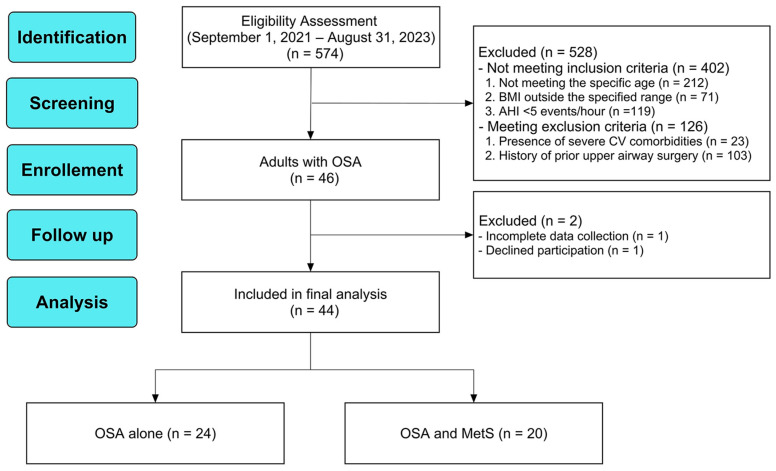
Flowchart of participant recruitment and selection. The reporting follows the Strengthening the Reporting of Observational Studies in Epidemiology (STROBE) guidelines [20]. Abbreviations: AHI, apnea–hypopnea index; BMI, body mass index; CV, cardiovascular; MetS, metabolic syndrome; OSA, obstructive sleep apnea.

From 1 September 2021, to 31 August 2023, adults aged 20 to 65 years who presented with habitual snoring at the Department of Otorhinolaryngology–Head and Neck Surgery were prospectively screened. Diagnosis of OSA was established according to the American Academy of Sleep Medicine (AASM) standards and corresponded to the International Classification of Diseases, 10th Revision (ICD-10) code G47.33. Inclusion criteria were aged 20 to 65 years, had a body mass index (BMI) between 18.5 and 40 kg/m^2^, and demonstrated an obstructive AHI of ≥5 events/h. Exclusion criteria included severe OSA accompanied by severe comorbidities requiring urgent care, morbid obesity (>40 kg/m^2^) necessitating immediate intervention, current or prior treatment for major psychiatric or cognitive disorders, and a history of carotid atherosclerosis, cardiovascular, metabolic, endocrine, inflammatory, or malignant diseases. Participant flow is illustrated in Figure 1.

### 2.3. Baseline Clinical and Anthropometric Measurements

Baseline assessments included BMI, neck circumference (NC), waist circumference (WC), and resting systolic and diastolic blood pressure (SBP/DBP) [21].

### 2.4. Pittsburgh Sleep Quality Index

Sleep quality was evaluated using the Pittsburgh Sleep Quality Index (PSQI), a validated self-report instrument covering a one-month period [22]. The PSQI comprises 19 items across seven components: overall sleep quality, sleep latency, sleep duration, sleep efficiency, sleep disturbances, need medications to sleep, and daytime dysfunction due to sleepiness. Each component is scored from 0 to 3, yielding a total score from 0 to 21; scores > 5 indicate poor sleep quality [23].

The PSQI has demonstrated acceptable internal consistency (Cronbach’s α = 0.70–0.83), stable test–retest reliability, and strong construct validity through correlations with objective sleep measures and its ability to differentiate good versus poor sleepers [24]. Its clinical utility and ease of administration make it a preferred tool in studies examining sleep and cardiometabolic risk in OSA populations [25,26].

### 2.5. Polysomnography

Participants underwent standard overnight, attended in-laboratory polysomnography supervised by registered sleep technologists. Sleep stages and respiratory events were manually scored according to the AASM Manual for the Scoring of Sleep and Associated Events (2017 guidelines) [27]. Key parameters recorded included AHI, apnea index (AI), 3% oxygen desaturation index (ODI3), mean peripheral oxygen saturation (SpO_2_), and minimum SpO_2_. For analytic purposes, severe OSA was strictly defined as an AHI ≥ 30 events/h.

### 2.6. Dietary Behavior Questionnaire

Dietary behaviors were assessed using the validated 12-item Dietary Behavior Questionnaire (DBQ), an instrument originally developed and validated for health promotion in Taiwanese populations [28]. The DBQ evaluates habitual dietary practices and modifiable lifestyle factors relevant to chronic disease prevention [29]. The questionnaire comprises two distinct domains: the dietary domain (Items 1–7), which focuses on nutritional balance and intake patterns (e.g., regular meal timing, sufficient fruit/vegetable intake, and avoidance of sugary or fried foods); and the behavioral domain (Items 8–12), which assesses eating mechanics and psychological cues (e.g., emotional eating, distracted eating, and shopping while hungry). In the current study cohort, the instrument demonstrated acceptable internal consistency (Cronbach’s *α* = 0.68).

Each item is rated on a four-point Likert scale ranging from 0 (“rarely”) to 3 (“always”). Items reflecting maladaptive behaviors (e.g., emotional eating) are reverse-scored, such that the total score ranges from 0 to 36, with higher scores indicating healthier overall dietary behaviors. For this study, particular attention was given to Item 3 (slow chewing (>20×): “*I chew slowly when eating (at least 20 times per bite).*” This item was utilized as a specific behavioral proxy for eating speed and mindful eating [30]. Based on established scoring thresholds, overall dietary behavior was stratified into four categories: poor (total score 0–12), fair (13–20), good (21–30), or excellent (31–36) (Supplementary Table S1).

### 2.7. International Physical Activity Questionnaire-Short Form

PA was evaluated using the validated Chinese short-form version of the International Physical Activity Questionnaire (IPAQ-SF) [31]. This self-report instrument estimates habitual PA across three intensity domains—walking, moderate-intensity, and vigorous-intensity activity—as well as daily sedentary time [32]. Participants reported the frequency (days per week) and duration (minutes per day) of activities performed over the preceding seven days. Data were converted into metabolic equivalent of task (MET) units using standard coefficients: walking = 3.3 METs, moderate activity = 4.0 METs, and vigorous activity = 8.0 METs. Total PA volume was calculated as the sum of walking, moderate, and vigorous MET-min/week [33]. To enhance clinical interpretability, these values were subsequently converted to MET-h/week (MET-min/week ÷ 60). PA levels were stratified into specific categories: Low PA was defined according to standard IPAQ protocols as failing to meet the criteria for moderate or high activity, specifically falling below 600 MET-min/week (10 MET-h/week) [33]. The IPAQ-SF has demonstrated robust psychometric properties [34], including high test–retest reliability (intraclass correlation coefficients of 0.76–0.88) [35] and construct validity [32] through correlations with accelerometer data and cardiovascular risk markers. The Chinese version has been culturally adapted and validated, ensuring consistent internal structure and responsiveness [36].

### 2.8. Dual-Energy X-Ray Absorptiometry

Baseline body composition was measured using DXA with a Hologic Delphi A scanner (Hologic Inc., Bedford, MA, USA). Scans were analyzed using Apex software (version 5.6), following standardized manufacturer protocols for participant positioning and region of interest placement [37]. Extracted parameters included total body fat percentage (TBF%), fat mass (FM), lean mass (LM), visceral adipose tissue (VAT), and appendicular lean mass index (ALM/height^2^), providing a comprehensive profile of adiposity and skeletal muscle distribution relevant to metabolic and sleep-related outcomes.

### 2.9. Fasting Laboratory Tests

After a minimum 12-h overnight fast, venous blood samples were collected between 7:00 AM and 10:00 AM [38]. Laboratory analyses included high-density lipoprotein cholesterol (HDL-C), low-density lipoprotein cholesterol (LDL-C), total cholesterol, triglycerides, fasting plasma glucose, glycohemoglobin, insulin, uric acid, and creatinine. Insulin resistance was calculated using the homeostatic model assessment (HOMA-IR) [39]:

HOMA−IR=Fasting InsulinμUmL×Fasting Glucose(mgdL)405.

This panel enabled evaluation of metabolic risk and MetS status in patients with OSA.

### 2.10. Definition of MetS

MetS was diagnosed according to the modified criteria for Asian populations [40]. Participants were classified as having MetS if they met at least three of the following five criteria: (1) Central obesity: WC ≥ 90 cm in men or ≥80 cm in women; (2) Hypertriglyceridemia: triglycerides ≥ 150 mg/dL; (3) Low HDL-C: <40 mg/dL in men or <50 mg/dL in women; (4) Elevated BP: systolic ≥ 130 mmHg or diastolic ≥ 85 mmHg; and (5) Hyperglycemia: fasting plasma glucose ≥ 100 mg/dL or current use of antidiabetic medication.

### 2.11. Outcome Measures

Primary outcomes included the presence of MetS, the seven component scores and global score of the PSQI for subjective sleep quality measurement, and the AHI, AI, ODI3, mean SpO_2_, and minimum SpO_2_ for objective sleep quality measurement. Secondary outcomes included the individual item scores, dietary score, behavior score, and total score of the DBQ, as well as the three physical activity domain scores and sedentary time derived from the IPAQ-SF. Supplementary outcomes included body composition metrics and fasting blood biochemical variables.

### 2.12. Sample Size Determination

The sample size for this exploratory study was primarily determined by the number of eligible patients meeting the strict inclusion criteria within the prospective recruitment window. To assess the statistical robustness of our findings, a post hoc power analysis was conducted using G*Power 3.1.9.7 (Heinrich Heine University, Düsseldorf, Germany). With a final sample size of 44 participants, the study achieved 80% power to detect a correlation coefficient of *r* ≥ 0.41 at a two-sided significance level (*α*) of 0.05. Consequently, the study is adequately powered to identify moderate-to-strong associations between lifestyle factors and clinical outcomes, while weaker associations should be interpreted with caution and require validation in larger cohorts.

### 2.13. Statistical Analysis

All statistical analyses were conducted using SPSS (version 29.0; IBM Corp., Armonk, NY, USA) and GraphPad Prism (version 10.3.0; GraphPad Software, Boston, MA, USA). Continuous variables were summarized as median and interquartile range (IQR), while categorical variables were reported as counts and percentages. Group comparisons were performed using non-parametric tests, including the Mann–Whitney *U* test, Chi-square test, and Fisher’s exact test, as appropriate. Spearman correlation was used to evaluate associations between variables. Given the exploratory nature of this study, no global adjustment for multiple comparisons (e.g., Bonferroni correction) was applied. Emphasis was placed on the magnitude of effect sizes and the consistency of association patterns rather than isolated statistical significance, to avoid inflating Type II error rates in this hypothesis-generating context [41].

Hierarchical logistic regression analyses were conducted to identify independent variables associated with MetS, poor sleep quality, and severe OSA. Prior to model construction, a “correct sign” approach was utilized to screen potential predictors, ensuring that the direction of association was biologically plausible [42]. Furthermore, multicollinearity among independent variables was rigorously assessed using variance inflation factors (VIF) [43]. All variables included in the final models exhibited VIF values < 10, indicating the absence of significant multicollinearity [44]. Results are presented as adjusted odds ratios (aORs) with 95% confidence intervals (CIs). A two-sided *p*-value < 0.05 was considered statistically significant.

## 3. Results

### 3.1. Patient Characteristics

A total of 574 patients were screened during the study period. Of these, 528 were excluded. The primary reasons for exclusion were not meeting the inclusion criteria (n = 402), predominantly due to individuals not meeting the specific age (n = 212), BMI outside the specified range (n = 71), or AHI < 5 events/h (n = 119). An additional 126 candidates were excluded based on specific exclusion criteria, such as the presence of severe cardiovascular comorbidities (n = 23) or a history of prior upper airway surgery for OSA (n = 103). Forty-six patients were initially enrolled, with two subsequently excluded due to incomplete data collection (n = 1) and withdrawal of consent (n = 1).

The final analytic sample comprised 44 adults with OSA, including 24 (55%) with OSA alone and 20 (45%) with comorbid MetS (Figure 1). The overall cohort had a mean age of 38.3 ± 9.1 years and was predominantly male (n = 39, 89%). The OSA with MetS group did not differ significantly from the OSA alone group in age or sex distribution (*p* > 0.05 for both). There were no significant differences in age or sex distribution between the two groups (*p* > 0.05). However, as detailed in Table 1, participants with comorbid MetS exhibited significantly greater central adiposity, characterized by a higher median BMI (27.5 vs. 24.4 kg/m^2^; *p* = 0.001) and waist circumference (104.5 vs. 98.0 cm; *p* = 0.004) compared to those with OSA alone.

### 3.2. Subjective and Objective Sleep Quality

In the overall cohort, the mean Global PSQI score was 8.2 ± 3.1, with 34 participants (77%) classified as having poor sleep quality (score > 5). Regarding objective severity, the mean AHI was 41.6 ± 27.4 events/h, and 25 participants (57%) were diagnosed with severe OSA (AHI ≥ 30 events/h).

Table 1 presents the comparison of subjective and objective sleep parameters between participants with OSA alone and those with comorbid MetS. Subjective sleep quality, as assessed by the Global PSQI score, was comparable between the two groups (*p* = 0.981). While the OSA with MetS group exhibited a trend toward higher component scores for sleep duration (median score: 1 vs. 0; *p* = 0.082)—suggesting shorter sleep duration—this difference did not reach statistical significance. The prevalence of poor sleep quality was similarly high in both groups (75% vs. 79%; *p* > 0.999).

In contrast, objective polysomnographic measures revealed significantly more pronounced nocturnal hypoxemia in the MetS group. Participants with OSA and MetS demonstrated a significantly higher ODI3 (median 45.9 vs. 17.0 events/h; *p* = 0.011) and lower mean SpO_2_ (93% vs. 96%; *p* = 0.009). Although the AHI was elevated in the OSA with MetS group, the difference approached but did not strictly reach statistical significance (median 47.2 vs. 26.6 events/h; *p* = 0.055). Similarly, while the proportion of severe OSA was higher in the OSA with MetS group compared to the OSA alone group (70% vs. 46%), this difference was not statistically significant (*p* = 0.135).

### 3.3. Dietary Behavior

Table 2 presents the distribution of dietary and behavioral scores derived from the DBQ. Generally, dietary habits were comparable between the two groups. No statistically significant differences were observed between participants with OSA alone and those with comorbid MetS across most item scores, aggregate dietary or behavioral scores, total DBQ scores, or dietary behavior classification (all *p* > 0.05). However, a notable exception was observed in eating mechanics: the score for slow chewing (>20×) was significantly lower in the OSA with MetS group compared to the OSA alone group (*p* = 0.047), indicating a tendency toward faster eating in patients with metabolic dysfunction. The overall quality of dietary behaviors was similar, with comparable proportions of participants categorized as having poor, fair, good, or excellent dietary behavior in both groups (*p* = 0.365).

### 3.4. Physical Activity

Table 2 summarizes the distribution of physical activity variables based on the IPAQ-SF. Despite the higher metabolic burden in the MetS group, no statistically significant differences were observed across any domain of weekly activity. Sedentary time was substantial in both groups (median 56.0 vs. 59.0 h/week; *p* = 0.260). Similarly, volume of walking, moderate-intensity, and vigorous-intensity activity (expressed in MET-h/week) did not differ significantly (*p* > 0.05). However, when stratified by activity level, there was a trend suggesting a higher prevalence of low PA in the OSA with MetS group (45%) compared to the OSA alone group (17%), although this difference did not strictly reach statistical significance (*p* = 0.053).

### 3.5. Body Composition and Blood Metabolic Profile

As shown in Table 3, participants with OSA and MetS exhibited significantly greater overall adiposity compared to those with OSA alone. Specifically, the MetS group had a significantly higher percentage of TBF (%) (median 34.4% vs. 30.1%; *p* = 0.009) and total FM (29,659 vs. 22,279 g; *p* = 0.001). While VAT mass was elevated in the MetS group (median 647 vs. 484 g), this difference approached but did not reach statistical significance (*p* = 0.075).

In terms of metabolic biomarkers, the MetS group demonstrated a distinct dyslipidemic profile, characterized by significantly higher triglyceride levels (median 179 vs. 107 mg/dL; *p* < 0.001) and lower HDL-C levels (42 vs. 49 mg/dL; *p* = 0.001). No statistically significant differences were observed between the groups for LDL-C, total cholesterol, fasting glucose, glycohemoglobin, insulin, or HOMA-IR (*p* > 0.05).

### 3.6. Correlations Between Lifestyle Behaviors and Metabolic Phenotypes

**Figure 2:**
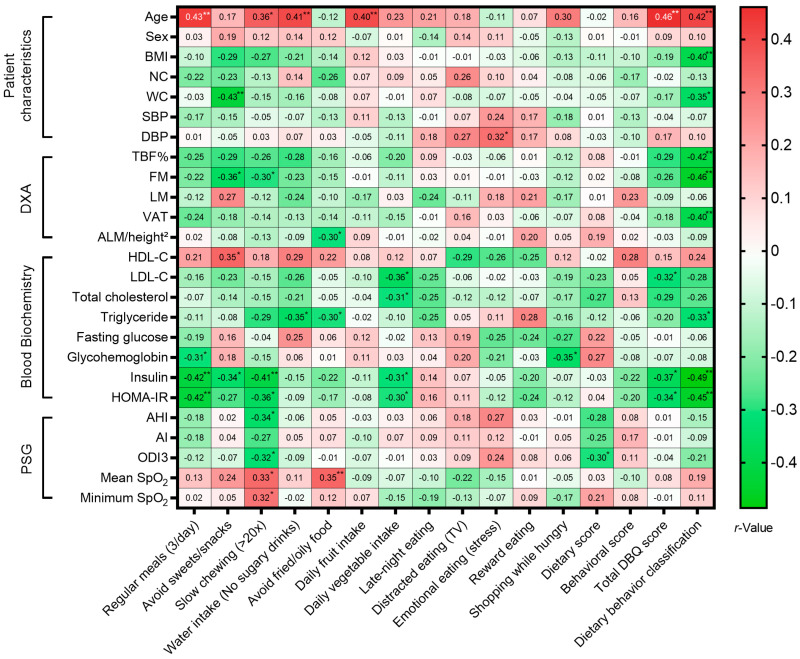
Spearman correlation matrix illustrating the associations between dietary behaviors (Dietary Behavior Questionnaire) and patient characteristics, body composition, metabolic biomarkers, and sleep parameters. The heat map shows correlation coefficients (*r*), displayed as black or white numbers within the boxes, with colors ranging from green (negative) to red (positive). Asterisks denote statistical significance (* *p* < 0.05, ** *p* < 0.01). Abbreviations: AHI, apnea–hypopnea index; AI, apnea index; ALM/height^2^, appendicular lean mass index; BMI, body mass index; DBP, diastolic blood pressure; DXA, dual-energy X-ray absorptiometry; FM, fat mass; HDL-C, high-density lipoprotein cholesterol; HOMA-IR, homeostatic model assessment for insulin resistance; LDL-C, low-density lipoprotein cholesterol; LM, lean mass; NC, neck circumference; ODI3, 3% oxygen desaturation index; PSG, polysomnography; SBP, systolic blood pressure; SpO_2_, peripheral oxygen saturation; TBF, total body fat; TV, television; VAT, visceral adipose tissue; WC, waist circumference.

To validate the physiological relevance of self-reported lifestyle behaviors, we analyzed their associations with objective metabolic, anthropometric, and polysomnographic markers (Figure 2). To minimize the risk of Type I error due to multiple comparisons, we conservatively report only associations reaching a significance threshold of *p* < 0.01.

Regarding dietary behaviors, the better dietary classification were strongly inversely associated with key markers of adiposity and insulin resistance, including BMI (*r* = −0.40), FM (*r* = −0.42), VAT (*r* = −0.40), fasting insulin (*r* = −0.48), and HOMA-IR (*r* = −0.45). Specific eating behaviors showed distinct metabolic footprints: Regular meals (3/day) was significantly inversely correlated with fasting insulin (*r* = −0.42) and HOMA-IR (*r* = −0.42) whereas slow chewing (>20 times/bite) was significantly inversely related with fasting insulin (*r* = −0.41). Additionally, the avoid sweets/snacks was inversely associated with WC (*r* = −0.43). Interestingly, healthier dietary habits—including regular meals, avoidance of sugary drinks, and daily fruit intake—were positively correlated with increasing age (*r* = 0.40–0.46), suggesting better dietary adherence among older participants.

**Figure 3:**
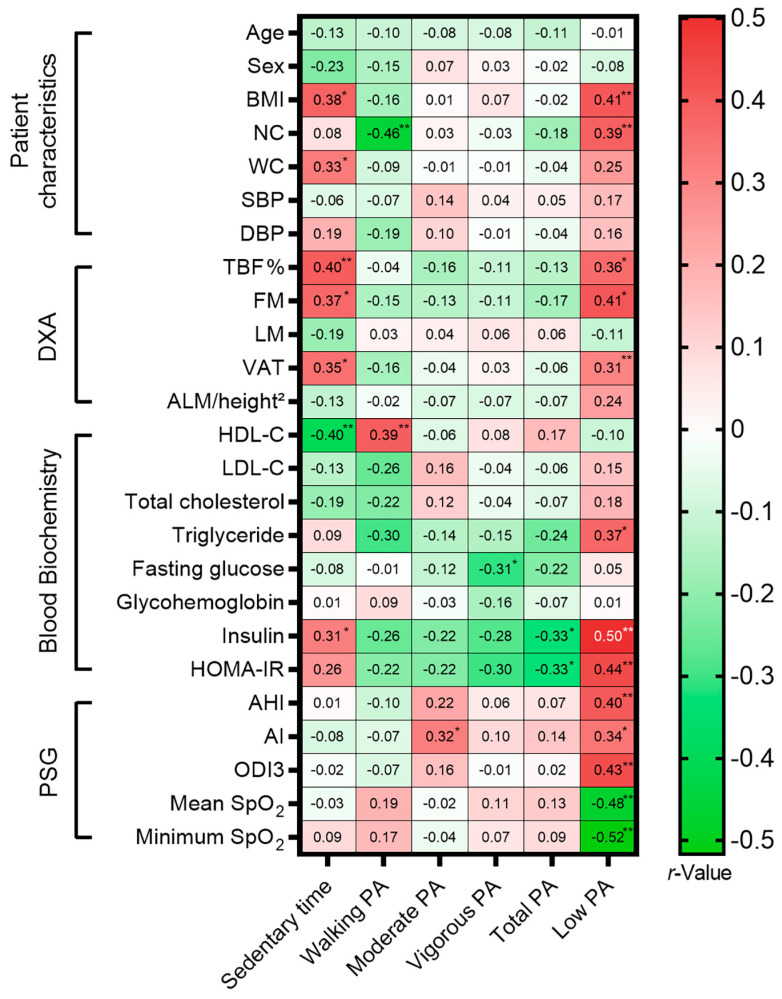
Spearman correlation matrix illustrating the associations between physical activity (PA) (International Physical Activity Questionnaire-Short Form) and patient characteristics, body composition, metabolic biomarkers, and sleep parameters. The heat map shows correlation coefficients (*r*), displayed as black or white numbers within the boxes, with colors ranging from green (negative) to red (positive). Asterisks denote statistical significance (* *p* < 0.05, ** *p* < 0.01). Abbreviations: AHI, apnea–hypopnea index; AI, apnea index; ALM/height^2^, appendicular lean mass index; BMI, body mass index; DBP, diastolic blood pressure; DXA, dual-energy X-ray absorptiometry; FM, fat mass; HDL-C, high-density lipoprotein cholesterol; HOMA-IR, homeostatic model assessment for insulin resistance; LDL-C, low-density lipoprotein cholesterol; LM, lean mass; NC, neck circumference; ODI3, 3% oxygen desaturation index; PSG, polysomnography; SBP, systolic blood pressure; SpO_2_, peripheral oxygen saturation; TBF, total body fat; VAT, visceral adipose tissue; WC, waist circumference.

Regarding PA (Figure 3), Sedentary time was positively associated with TBF% (*r* = 0.40) and inversely related to HDL-C (*r* = −0.40). Conversely, total walking PA was associated with a favorable metabolic profile, including higher HDL-C (*r* = 0.39) and reduced NC (*r* = −0.46). Notably, low PA status was strongly linked not only to metabolic dysfunction (higher BMI, insulin, and HOMA-IR) but also to worse respiratory severity. Specifically, low PA was positively associated with AHI (*r* = 0.40) and ODI3 (*r* = 0.43), and strongly inversely correlated with mean SpO_2_ (*r* = −0.48) and minimum SpO_2_ (*r* = −0.52), underscoring the link between inactive PA and nocturnal hypoxemia.

### 3.7. Factors Related to MetS, Poor Sleep Quality, and Severe OSA

**Figure 4:**
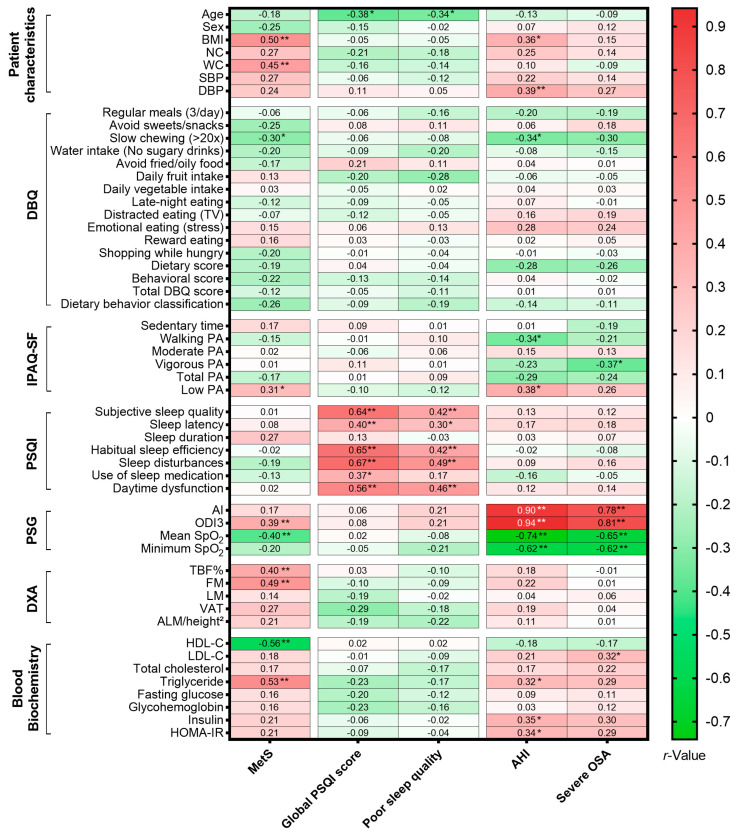
Correlation heatmap of lifestyle behaviors, sleep metrics, and metabolic markers. The color gradient illustrates the strength and direction of the Spearman correlation coefficient (*r*), ranging from green (negative) to red (positive). The black or white numbers within the cells indicate the specific *r* values. Statistical significance is denoted by asterisks (* *p* < 0.05; ** *p* < 0.01). Notably, significant correlations did not imply causation and should be interpreted within the broader clinical and physiological context. Abbreviations: AHI, apnea–hypopnea index; AI, apnea index; ALM/height^2^, appendicular lean mass index; BMI, body mass index; DBQ, Dietary Behavior Questionnaire; DBP, diastolic blood pressure; DXA, dual-energy X-ray absorptiometry; FM, fat mass; HDL-C, high-density lipoprotein cholesterol; HOMA-IR, homeostatic model assessment for insulin resistance; IPAQ-SF, International Physical Activity Questionnaire-Short Form; LDL-C, low-density lipoprotein cholesterol; LM, lean mass; MetS, metabolic syndrome; NC, neck circumference; ODI3, 3% oxygen desaturation index; PA, physical activity; PSG, polysomnography; PSQI, Pittsburgh Sleep Quality Index; SBP, systolic blood pressure; SpO_2_, peripheral oxygen saturation; TBF, total body fat; TV, television; VAT, visceral adipose tissue; WC, waist circumference.

Spearman correlation analysis was performed to identify significant associations between the three primary outcomes—MetS, poor sleep quality, and severe OSA—and various lifestyle behaviors, sleep metrics, and metabolic indicators (Figure 4).

#### 3.7.1. Factors Associated with MetS

MetS was strongly positively correlated with indices of adiposity, including BMI (*r* = 0.50; *p* < 0.001), WC (*r* = 0.45; *p* = 0.002), TBF (%) (*r* = 0.40; *p* = 0.007), and FM (*r* = 0.49; *p* < 0.001). As expected by definition, MetS participants had significantly higher triglycerides (*r* = 0.53; *p* < 0.001) and lower HDL-C (*r* = −0.56; *p* < 0.001). Regarding lifestyle factors, slow chewing (>20×) was significantly less common in the MetS group (*r* = −0.30; *p* = 0.046), suggesting a link between rapid eating behavior and metabolic dysfunction. Furthermore, MetS was significantly associated with low PA (*r* = 0.31; *p* = 0.041).

#### 3.7.2. Factors Associated with Poor Sleep Quality

The analysis revealed a distinct dissociation between subjective sleep complaints and objective metabolic or respiratory parameters. Age emerged as the only significant predictor of both global PSQI scores and poor sleep quality (*r* = −0.38; *p* = 0.010; *r* = −0.34; *p* = 0.025), indicating that younger participants in this cohort reported worse subjective sleep. No significant associations were observed between poor sleep quality and anthropometric measures, dietary behaviors, polysomnographic parameters, or blood biochemical markers.

#### 3.7.3. Factors Associated with Severe OSA

Severe OSA demonstrated specific metabolic and lifestyle associations distinct from generalized obesity. It was significantly positively correlated with LDL-C (*r* = 0.32; *p* = 0.038) and inversely related to vigorous PA (*r* = −0.37; *p* = 0.014), highlighting the role of exercise intensity in disease severity. Additionally, AHI was positively associated with BMI (*r* = 0.36; *p* = 0.015), DBP (*r* = 0.39; *p* = 0.008), low PA (*r* = 0.38; *p* = 0.010), triglyceride (*r* = 0.32; *p* = 0.032), insulin level (*r* = 0.35; *p* = 0.019), and HOMA-IR (*r* = 0.34; *p* = 0.026), and inversely correlated with slow chewing (*r* = −0.34; *p* = 0.026), walking PA (*r* = −0.34; *p* = 0.023). No significant correlations were observed between severe OSA and other general dietary scores or body composition markers (DXA components).

### 3.8. Independent Variables Associated with MetS, Poor Sleep Quality, and Severe OSA

Hierarchical logistic regression analyses were performed to identify independent variables associated with MetS (Table 4), poor sleep quality (Table 5), and severe OSA (Table 6).

#### 3.8.1. Metabolic Syndrome

In the analysis for MetS (Table 4), Model 1 (demographics and BMI) identified BMI as a significant independent correlate (aOR = 1.44, 95% CI 1.11–1.87; *p* = 0.006). This association remained robust after the addition of dietary behaviors in Model 2. When PA parameters were introduced in Model 3, both BMI (aOR = 1.56, 95% CI 1.14–2.13; *p* = 0.005) and total PA (aOR = 0.97, 95% CI 0.94–0.99; *p* = 0.037) were significant independent variables. In the fully adjusted Model 4, higher BMI (aOR = 1.64; *p* = 0.008) and reward eating (aOR = 3.34, 95% CI 1.05–10.63; *p* = 0.041) were independently associated with increased odds of MetS, while higher total PA (aOR = 0.96; *p* = 0.026) was associated with reduced odds. Interestingly, moderate PA was positively associated with MetS in this adjusted model (aOR = 1.09; *p* = 0.041). However, it is important to note that this final model operates with a low events-per-variable (EPV) ratio of 5 (20 patients with MetS and 4 variables) due to sample size constraints [45]. Further, the Hosmer–Lemeshow test (*χ*^2^ = 14.22, *p* = 0.076) indicated acceptable fit for the multivariable logistic regression model [46]. Consequently, these specific multivariable findings should be interpreted as exploratory and hypothesis-generating, pending replication in larger datasets to address statistical power limitations.

#### 3.8.2. Poor Sleep Quality

Regarding poor sleep quality (Table 5), Model 1 identified age as the sole independent factor, where each additional year was associated with a 9% reduction in the odds of reporting poor sleep (aOR = 0.91, 95% CI 0.83–0.99; *p* = 0.037). This association persisted across all subsequent models (Models 2–4) after adjusting for dietary and PA behaviors. With 34 participants classified as having poor sleep quality and age serving as the primary variable, this analysis maintains a high EPV ratio (>30). The non-significant Hosmer–Lemeshow test further confirms model fit and estimate stability.

#### 3.8.3. Severe OSA

In the hierarchical analysis for severe OSA (Table 6), demographic factors in Model 1 (age, sex, BMI) were not statistically significant. However, the addition of dietary behaviors in Model 2 revealed that slow chewing (aOR = 0.27, 95% CI 0.09–0.82; *p* = 0.021) and emotional eating (aOR = 2.27, 95% CI 1.03–5.01; *p* = 0.043) were significant independent variables. While Model 3 identified associations with moderate PA (*p* = 0.040) and vigorous PA (*p* = 0.048), these PA variables lost statistical significance in the final model. In the fully adjusted Model 4, slow chewing (aOR = 0.24, 95% CI 0.06–0.92; *p* = 0.038) and emotional eating (aOR = 2.40, 95% CI 1.01–5.72; *p* = 0.048) remained the two significant independent variables associated with severe OSA, even after controlling for age, male sex, BMI, moderate PA, and vigorous PA. With 25 participants classified as having severe OSA and two primary variables identified, this model satisfies the recommended EPV of 10, suggesting adequate reliability for these associations despite the limited total sample size. The non-significant Hosmer–Lemeshow test confirms adequate fit and stability.

## 4. Discussion

This exploratory study underscores the complex interplay between lifestyle behaviors, metabolic dysfunction, and sleep-disordered breathing. In a cohort of predominantly young males with OSA, we observed a high prevalence of MetS (45%). Notably, although the frequency of respiratory events (AHI) did not differ between groups, participants with comorbid MetS exhibited markedly more severe nocturnal hypoxemia—reflected by higher ODI3 values and lower SpO_2_—highlighting that hypoxemia can occur independently of event frequency [47]. This dissociation suggests that central adiposity may amplify the physiological consequences of apnea; mechanistically, excessive visceral fat (indexed by elevated waist circumference) likely reduces functional residual capacity and end-expiratory lung volume, resulting in more rapid oxyhemoglobin desaturation during respiratory events [48].

Beyond physiological markers, the most novel finding of this investigation emerged from the hierarchical regression analyses: psychological eating behaviors were identified as a robust, independent variable associated with MetS, exceeding the explanatory value of traditional demographic and anthropometric markers such as age, sex, and BMI [49]. Specifically, emotional eating was significantly associated with increased odds of MetS even after strict adjustment for BMI and PA. Emotional eating may theoretically be linked to metabolic dysregulation through mechanisms not fully captured by overall adiposity or activity levels, including stress-related hormonal responses [50], late-night high-calorie intake [51], and greater glycemic and lipid variability [52]. The persistence of this association after controlling for BMI and exercise suggests that the underlying pattern and motivation of eating behavior—such as emotional eating—may be associated with cardiometabolic risk beyond simple energy balance [53]. Incorporating emotional-eating screening into OSA clinics may therefore help identify patients at elevated metabolic risk who could potentially benefit from targeted psychological or behavioral interventions alongside standard lifestyle counseling and CPAP therapy [54]. Integrating structured support, such as cognitive-behavioral strategies for emotion regulation and eating, may represent a promising approach to addressing MetS in this population [55].

From a behavioral standpoint, this study is also among the first to identify reward eating as a strong, independent correlate of MetS in an OSA cohort. Unlike homeostatic hunger, reward eating is characterized by consumption driven by hedonic cues and emotional regulation [55]. This finding aligns with neurobehavioral evidence suggesting that sleep fragmentation and intermittent hypoxia may disrupt hypothalamic–mesolimbic reward pathways, potentially increasing the drive for hyper-palatable, energy-dense foods [56,57]. Together, these results support a hypothesized “vicious cycle” in which OSA-related fatigue and neurohormonal alterations may promote hedonic eating, which in turn could exacerbate the central adiposity and insulin resistance characteristic of MetS.

Furthermore, our models confirmed the inverse association of total PA with MetS, consistent with evidence that overall activity—including light and vigorous intensities—is associated with lower MetS prevalence [58]. In contrast, moderate PA was positively associated with MetS, a finding that diverges from the dominant literature showing inverse associations with moderate-to-vigorous activity [59]. No prior studies have reported increased odds of MetS associated with isolated moderate PA; instead, favorable outcomes typically emerge in dose–response patterns. This apparent paradox may reflect measurement limitations, reverse causation inherent to cross-sectional designs, or context-specific factors such as reliance on moderate activity without vigorous components [57]. Future research should employ prospective or interventional designs with repeated assessments of PA and MetS to clarify temporal relationships. Studies should integrate device-based and domain-specific PA measures (light, moderate, vigorous; leisure vs. occupational) and model intensities jointly using dose–response and substitution frameworks (e.g., replacing moderate with vigorous activity) to elucidate why moderate PA appears positively associated while total PA remains inversely associated [60,61].

Another intriguing finding was the inverse relationship between age and subjective sleep quality, with younger participants reporting significantly higher PSQI scores. This deviates from general epidemiological trends where sleep quality typically deteriorates with aging [62]. However, in the context of this relatively young, working-age cohort, this likely reflects the distinct pressures of modern lifestyle factors [63]. Younger adults are more susceptible to social jetlag, bedtime procrastination, and excessive screen time exposure—behaviors that disrupt circadian rhythms and heighten pre-sleep arousal [64]. Furthermore, this finding reinforces the “phenotypic mismatch” often observed in clinical OSA populations: younger patients frequently present with an “insomnia-like” phenotype characterized by lower arousal thresholds and higher sympathetic reactivity, leading to greater subjective sleep dissatisfaction [65]. In contrast, older adults with long-standing OSA may develop a tolerance to sleep fragmentation or present primarily with excessive daytime sleepiness rather than perceived poor sleep quality [66]. This dissociation implies that in younger OSA patients, standard metrics like AHI may fail to capture the full burden of the disorder, necessitating a broader assessment of psychological and behavioral sleep disruptors.

Perhaps the most clinically relevant finding of this study is the identification of specific eating mechanics as independent correlates of OSA severity, distinct from the influence of generalized obesity. We observed that “slow chewing” was associated with significantly reduced odds of severe OSA, whereas “emotional eating” was associated with more than double the odds. Mechanistically, rapid eating is known to disrupt the gut–brain satiety cascade, potentially contributing to delayed postprandial hormone release and subsequent caloric overconsumption [6,67,68]. It is hypothesized that by chewing slowly, patients may experience enhanced sensory satiety and glycemic control, potentially mitigating the metabolic strain associated with OSA [69]. Furthermore, from an anatomical perspective, rigorous and prolonged mastication recruits the muscles of mastication and the tongue (specifically the genioglossus) [8], theoretically acting as a form of functional training [9]. This aligns with the principles of orofacial myofunctional therapy, where increased tone in upper airway dilator muscles has been shown to reduce airway collapsibility and AHI severity [70]. Thus, slow chewing may theoretically be linked to improved outcomes via two pathways: metabolic regulation via improved satiety signaling and mechanical stabilization of the upper airway via increased muscle tone [71]. Conversely, the strong link between emotional eating and severe OSA suggests a bidirectional neurobehavioral pathway [72,73]. Chronic sleep fragmentation and intermittent hypoxia impair prefrontal cortex function—the center of impulse control—while simultaneously activating the limbic reward system [74,75]. This neurocognitive disinhibition renders patients more susceptible to stress-induced eating, potentially creating a “vicious cycle” where sleep loss exacerbates maladaptive dietary behaviors that further entrench metabolic and respiratory dysfunction [76].

Regarding PA, our univariate analysis indicated that vigorous-intensity PA was inversely associated with severe OSA and insulin resistance markers, highlighting the importance of exercise intensity over mere duration [77]. However, in our fully adjusted hierarchical model, the statistical significance of PA was attenuated when concurrent eating behaviors were included. This suggests that while exercise is beneficial for metabolic health [78], maladaptive eating behaviors (such as emotional eating and rapid consumption) may exert a more immediate and dominant influence on OSA severity in this population [72]. Consequently, therapeutic interventions should not rely solely on exercise prescription but must prioritize behavioral modification of eating habits [79]—specifically targeting eating speed and emotional regulation—to achieve optimal disease management.

This study has several strengths, including its prospective participant recruitment and comprehensive, multidimensional assessments of sleep, diet, PA, body composition, and metabolic biomarkers. However, several limitations must be acknowledged.

First, the cross-sectional design precludes causal inferences; specifically, we cannot determine whether healthy lifestyle behaviors reduce OSA severity or if milder disease severity simply enables better adherence to healthy behaviors. Second, the directionality of the associations regarding PA is complex; severe OSA-induced fatigue and excessive daytime sleepiness may limit a patient’s functional capacity to engage in exercise (“reverse causality”). Third, reliance on self-reported measures for PA (IPAQ-SF) and dietary behaviors (DBQ) introduces the potential for recall bias and social desirability bias, where participants may overreport “virtuous” behaviors (e.g., exercise duration) or underreport maladaptive eating habits [80].

Fourth, the modest sample size (n = 44) limits generalizability and statistical power. A sensitivity power analysis indicated that our sample provided 80% power to detect moderate-to-strong correlations (*r* = 0.41), suggesting that weaker associations may have been missed (Type II error). Additionally, given the number of exploratory correlation analyses performed (Figure 2, Figure 3 and Figure 4), there is an inherent risk of Type I error due to multiple comparisons. Therefore, these findings should be interpreted as exploratory and hypothesis-generating rather than definitive. Regarding the regression analyses, the multivariable model for MetS operates with a low EPV ratio (~5) [45], increasing the risk of overfitting. In contrast, the models for poor sleep quality and severe OSA met standard EPV criteria (>30 and >10, respectively), supporting greater statistical stability for those specific estimates.

Fifth, as a single-center study conducted at a tertiary referral hospital in Taiwan, our findings may be subject to selection bias and may not be fully representative of community-based populations or non-Asian ethnicities. Finally, the study cohort demonstrated a male predominance (~90%), which reflects the typical epidemiology of clinical OSA populations but limits the generalizability of our findings to female patients. Women with OSA often present with distinct phenotypic characteristics and metabolic risk profiles which our study may not fully capture.

Future research should prioritize longitudinal or randomized controlled trials to determine whether structured behavioral interventions—specifically slow chewing protocols—can causally reduce AHI or metabolic risk. Objective PA monitoring, such as accelerometry, will be essential to quantify dose–response relationships, and mechanistic studies are warranted to explore how walking influences airway anatomy and fluid dynamics.

## 5. Conclusions

This hypothesis-generating study identifies a distinct high-risk phenotype in young adults with OSA, characterized by MetS, severe nocturnal hypoxemia, and maladaptive lifestyle behaviors. Our preliminary findings suggest that traditional management—focused primarily on CPAP and generic weight loss—may be insufficient if the underlying behavioral drivers are ignored. Specifically, we observed that eating mechanics (fast eating) and psychological cues (emotional and reward eating) were robust, independent correlates of OSA severity, while higher total PA was associated with a lower prevalence of metabolic syndrome. Consequently, clinical management may benefit from a multidisciplinary model that integrates sleep medicine with behavioral nutrition and exercise physiology, emphasizing strategies to encourage slower eating speed, regulate emotional feeding, and reduce sedentary behavior. Future research should prioritize randomized controlled trials to determine whether modifying these specific lifestyle targets can causally interrupt the “vicious cycle” of metabolic and respiratory dysfunction in OSA. Such studies should utilize objective endpoints, including actigraphy and polysomnography, to validate these preliminary associations.

## Figures and Tables

**Table 1:** Demographic, anthropometric, blood pressure, Pittsburgh Sleep Quality Index and polysomnographic characteristics of participants by MetS status.

	Variables	OSA Alone (n = 24)	OSA with MetS (n = 20)	*p* ^a^
Demographics	Age, y	38 (32–45)	35 (30–44)	0.243
	Sex			0.160
	Men, n (%)	23 (96)	16 (80)	
	Women, n (%)	1 (4)	4 (20)	
Anthropometrics	BMI, kg/m^2^	**24.4 (23.1–26.1)**	**27.5 (26.3–29.6)**	**0.001**
	NC, cm	38.0 (37.0–41.0)	40.0 (39.0–41.0)	0.074
	WC, cm	**98.0 (95.3–102.0)**	**104.5 (100.9–109.0)**	**0.003**
Blood pressure	SBP, mmHg	131 (120–138)	138 (132–150)	0.073
	DBP, mmHg	78 (75–84.5)	82 (80–91)	0.114
Pittsburgh Sleep Quality Index	Subjective sleep quality, score	2 (1–3)	2 (2–3)	>0.999
	Sleep latency, score	2 (0–2)	2 (0–2)	0.611
	Sleep duration, score	0 (0–1)	1 (0–1)	0.082
	Habitual sleep efficiency, score	1 (0–2)	0 (0–2)	0.898
	Sleep disturbances, score	2 (1–2)	1 (1–2)	0.208
	Use of sleep medication, score	0 (0–0)	0 (0–0)	0.382
	Daytime dysfunction, score	1 (1–2)	1 (1–2)	0.891
	Global PSQI score	8 (6–10)	7 (5–11)	0.981
	Poor sleep quality (global PSQI score > 5), n (%)	19 (79)	15 (75)	>0.999
Polysomnography	AHI, events/h	26.6 (10.2–58.8)	47.2 (29.4–66.3)	0.055
	AI, events/h	7.9 (2.2–31.8)	14.4 (6.3–37.3)	0.268
	ODI3, events/h	**17.0 (3.7–37.0)**	**45.9 (29.5–62.5)**	**0.011**
	Mean SpO_2_, %	**96 (94–97)**	**93 (92–95)**	**0.009**
	Minimum SpO_2_, %	81 (75–90)	79 (72–84)	0.194
	Severe OSA (AHI ≥ 30 events/h), n (%)	11 (46%)	14 (70%)	0.135

Data are presented as median (interquartile range) or n (%). ^a^ *p* values were calculated using the Mann–Whitney *U* test for continuous variables and Fisher’s exact test for categorical variables. Bold values indicate statistical significance (*p* < 0.05). Abbreviations: AHI, apnea–hypopnea index; BMI, body mass index; DBP, diastolic blood pressure; MetS, metabolic syndrome; NC, neck circumference; ODI3, 3% oxygen desaturation index; OSA, obstructive sleep apnea; SBP, systolic blood pressure; SpO_2_, peripheral oxygen saturation; WC, waist circumference.

**Table 2:** Comparison of variables of the Dietary Behavior Questionnaire and International Physical Activity Questionnaire-Short Form of participants by MetS status.

Variables	OSA Alone (n = 24)	OSA with MetS (n = 20)	*p* ^a^
Dietary Behavior Questionnaire			
Regular meals (3/day)	2 (1–3)	2 (1–3)	0.702
Avoid sweets/snacks	2 (1–2)	1 (1–2)	0.100
Slow chewing (>20×)	**0 (1–1)**	**0 (0–1)**	**0.047**
Water intake (No sugary drinks)	2 (1–3)	1 (1–2)	0.183
Avoid fried/oily food	1 (1–2)	1 (1–2)	0.277
Daily fruit intake	1 (1–2)	2 (0–3)	0.398
Daily vegetable intake	2 (1–3)	2 (1–3)	0.830
Late-night eating	2 (2–3)	2 (2–3)	0.417
Distracted eating (TV)	2 (1–2)	2 (1–3)	0.637
Emotional eating (stress)	2 (1–3)	2 (2–3)	0.311
Reward eating	1 (2–2)	2 (1–3)	0.287
Shopping while hungry	2 (1–2)	2 (1–2)	0.186
Dietary score	11 (9–13)	10 (6–14)	0.205
Behavioral score	11 (9–12)	9 (7–11)	0.153
Total DBQ score	21 (17–24)	19 (17–22)	0.435
Dietary behavior classification			0.365
Poor, n (%)	1 (4)	2 (10)	
Fair, n (%)	10 (42)	12 (60)	
Good, n (%)	12 (50)	6 (30)	
Excellent, n (%)	1 (4)	0 (0)	
International Physical Activity Questionnaire-Short Form			
Sedentary time, h/week	56.0 (41.3–64.3)	59.0 (42.0–70.0)	0.255
Walking PA, MET-h/week	8.3 (1.1–15.4)	3.3 (1.7–7.8)	0.339
Moderate PA, MET-h/week	2.0 (0.2–7.5)	3.3 (0–13.5)	0.924
Vigorous PA, MET-h/week	5.3 (0.3–30.0)	6.5 (1.7–21.0)	0.962
Total PA, MET-h/week	23.1 (11.4–49.6)	18.2 (6.9–34.7)	0.263
PA classification			0.053
High, n (%)	20 (83)	11 (55)	
Low, n (%)	4 (17)	9 (45)	

Data are presented as median (interquartile range) or n (%). ^a^ *p* values were calculated using the Mann–Whitney *U* test for continuous variables and Chi-square test or Fisher’s exact test for categorical variables as appropriate. Bold values indicate statistical significance (*p* < 0.05). Abbreviations: MET, metabolic equivalent of task; MetS, metabolic syndrome; OSA, obstructive sleep apnea; PA, physical activity.

**Table 3:** Comparison of body composition and fasting blood biochemistry between OSA alone and OSA with MetS.

Measurements	Variables	OSA Alone (n = 24)	OSA with MetS (n = 20)	*p*-Value ^a^
Body composition	TBF (%)	**30.1 (27.6–32.9)**	**34.4 (31.7–36.7)**	**0.009**
	FM, g	**22** **,** **279 (17** **,** **484–25** **,** **055)**	**29** **,** **659 (24** **,** **276–32** **,** **392)**	**0.001**
	LM, g	47,873 (45,466–51,647)	49,798 (42,347–56,129)	0.370
	VAT, g	484 (443–627)	647 (518–757)	0.075
	ALM/height^2^; kg/m^2^	7.14 (6.82–7.75)	7.62 (6.96–8.41)	0.168
Fasting blood biochemistry	HDL-C, mg/dL	**49 (45–54.25)**	**42 (38–44)**	**<0.001**
	LDL-C, mg/dL	122 (105–145)	139 (112–148)	0.229
	Total cholesterol, mg/dL	196 (178–209)	209 (183–222)	0.263
	Triglyceride, mg/dL	**107 (80–128)**	**179 (132–208)**	**<0.001**
	Fasting glucose, mg/dL	90 (88–95)	93 (85–99)	0.290
	Glycohemoglobin, %	5.6 (5.4–5.9)	5.7 (5.5–6.1)	0.290
	Insulin, uU/mL	6.8 (4.18–9.0)	8.3 (5.6–10.2)	0.175
	HOMA-IR	1.54 (0.94–2.17)	1.94 (1.42–2.48)	0.164

Data are presented as median (interquartile range). ^a^ *p* values were calculated using the Mann–Whitney *U* test. Bold values indicate statistical significance (*p* < 0.05). Abbreviations: ALM/height^2^, appendicular lean mass index; FM, fat mass; HDL-C, high-density lipoprotein cholesterol; HOMA-IR, homeostatic model assessment for insulin resistance; LDL-C, low-density lipoprotein cholesterol; LM, lean mass; MetS, metabolic syndrome; OSA, obstructive sleep apnea; TBF, total body fat; VAT, visceral adipose tissue.

**Table 4:** Hierarchical logistic regression for metabolic syndrome.

	Model 1		Model 2		Model 3		Model 4 ^a^	
	(Demographics & BMI)		(Add Dietary Behavior)		(Add Physical Activity)		(Add Lifestyle)	
Variables	aOR (95% CI)	*p*	aOR (95% CI)	*p*	aOR (95% CI)	*p*	aOR (95% CI)	*p*
Step 1								
Age	0.96 (0.88–1.04)	0.266	0.97 (0.89–1.06)	0.483	0.94 (0.86–1.03)	0.212	0.95 (0.86–1.05)	0.334
Sex (male)	0.20 (0.02–2.34)	0.201	0.15 (0.01–3.28)	0.228	0.08 (0.01–1.55)	0.096	0.03 (0.01–1.53)	0.081
BMI	**1.44 (1.11–1.87)**	**0.006**	**1.41 (1.08–1.85)**	**0.012**	**1.56 (1.14–2.13)**	**0.005**	**1.64 (1.14–2.36)**	**0.008**
Step 2								
Slow chewing (>20×)	—	—	0.54 (0.17–1.56)	0.238				
Reward eating	—	—	2.29 (0.86–6.11)	0.097				
Step 3								
Moderate PA	—	—	—	—	1.07 (0.99–1.15)	0.066		
Total PA	—	—	—	—	**0.97 (0.94–0.99)**	**0.037**		
Step 4								
Slow chewing (>20×)	—	—	—	—	—	—	0.72 (0.19–2.70)	0.631
Reward eating	—	—	—	—	—	—	**3.34 (1.05–10.63)**	**0.041**
Moderate PA	—	—	—	—	—	—	**1.09 (1.00–1.19)**	**0.041**
Total PA	—	—	—	—	—	—	**0.96 (0.93–0.99)**	**0.026**

Bold values indicate statistical significance (*p* < 0.05). ^a^ Model diagnostics: Number of events = 20; Events per variable = 4. Hosmer–Lemeshow test for goodness-of-fit: *χ*^2^ = 14.22, *p* = 0.076. Abbreviations: aOR, adjusted odds ration; BMI, body mass index; CI, confidence interval; PA, physical activity.

**Table 5:** Hierarchical logistic regression for poor sleep quality.

	Model 1		Model 2		Model 3		Model 4 ^a^	
	(Demographics & BMI)		(Add Dietary Behavior)		(Add Physical Activity)		(Add Lifestyle)	
Variables	aOR (95% CI)	*p*	aOR (95% CI)	*p*	aOR (95% CI)	*p*	aOR (95% CI)	*p*
Step 1								
Age	**0.91 (0.83–0.99)**	**0.037**	**0.91 (0.82–0.99)**	**0.049**	**0.88 (0.79–0.98)**	**0.023**	**0.88 (0.78–0.99)**	**0.037**
Sex (male)	0.92 (0.08–10.21)	0.943	0.78 (0.07–9.03)	0.844	1.10 (0.09–13.17)	0.940	0.97 (0.07–12.77)	0.098
BMI	0.93 (0.73–1.18)	0.523	0.93 (0.72–1.20)	0.573	1.02 (0.78–1.36)	0.878	1.04 (0.78–1.38)	0.806
Step 2								
Slow chewing (>20×)	—	—	0.98 (0.33–2.91)	0.977				
Emotional eating (stress)	—	—	1.45 (0.64–3.29)	0.371				
Step 3								
Walking PA	—	—	—	—	1.14 (0.97–1.36)	0.121		
Moderate PA	—	—	—	—	1.03 (0.96–1.09)	0.436		
Step 4								
Slow chewing (>20×)	—	—	—	—	—	—	0.67 (0.19–2.34)	0.528
Emotional eating (stress)	—	—	—	—	—	—	1.71 (0.72–4.06)	0.228
Walking PA	—	—	—	—	—	—	1.19 (0.97–1.46)	0.088
Moderate PA	—	—	—	—	—	—	1.03 (0.96–1.09)	0.443

Bold values indicate statistical significance (*p* < 0.05). ^a^ Model diagnostics: Number of events = 34; Events per variable = 34. Hosmer–Lemeshow test for goodness-of-fit: *χ*^2^ = 10.59, *p* = 0.226. Abbreviations: aOR, adjusted odds ration; BMI, body mass index; CI, confidence interval; PA, physical activity.

**Table 6:** Hierarchical logistic regression for severe obstructive sleep apnea.

	Model 1		Model 2		Model 3		Model 4 ^a^	
	(Demographics & BMI)		(Add Dietary Behavior)		(Add Physical Activity)		(Add Lifestyle)	
Variables	aOR (95% CI)	*p*	aOR (95% CI)	*p*	aOR (95% CI)	*p*	aOR (95% CI)	*p*
Step 1								
Age	0.98 (0.92–1.05)	0.557	1.03 (0.94–1.11)	0.566	0.98 (0.91–1.06)	0.626	1.024 (0.937–1.119)	0.605
Sex (male)	2.41 (0.35–16.63)	0.373	3.67 (0.39–34.9)	0.257	2.46 (0.29–20.82)	0.409	4.695 (0.440–50.126)	0.201
BMI	1.07 (0.88–1.31)	0.505	1.02 (0.82–1.28)	0.836	1.094 (0.87–1.38)	0.453	1.044 (0.793–1.375)	0.756
Step 2								
Slow chewing (>20×)	—	—	**0.27 (0.09–0.82)**	**0.021**				
Emotional eating (stress)	—	—	**2.27 (1.03–5.01)**	**0.043**				
Step 3								
Moderate PA	—	—	—	—	**1.14 (1.01–1.30)**	**0.040**		
Vigorous PA	—	—	—	—	**0.94 (0.88–0.99)**	**0.048**		
Step 4								
Slow chewing (>20×)	—	—	—	—	—	—	**0.24 (0.06–0.92)**	**0.038**
Emotional eating (stress)	—	—	—	—	—	—	**2.40 (1.01–5.72)**	**0.048**
Moderate PA	—	—	—	—	—	—	1.10 (0.99–1.22)	0.053
Vigorous PA	—	—	—	—	—	—	0.95 (0.90–1.00)	0.064

Bold values indicate statistical significance (*p* < 0.05). ^a^ Model diagnostics: Number of events = 25; Events per variable = 2. Hosmer–Lemeshow test for goodness-of-fit: *χ*^2^ = 12.10, *p* = 0.147. Abbreviations: aOR, adjusted odds ration; BMI, body mass index; CI, confidence interval; PA, physical activity.

## Data Availability

The data presented in this study are available on request from the corresponding author due to ethical reasons.

## Supplementary Materials

The following supporting information can be downloaded at: https://www.mdpi.com/article/10.3390/nu18030409/s1, Table S1: Dietary Behavior Questionnaire. References [28,29,81] are cited in Supplementary Materials.

## Abbreviations

AASM	American Academy of Sleep Medicine
AHI	Apnea–hypopnea index
AI	Apnea index
ALM/height^2^	Appendicular lean mass index
aOR	Adjusted odds ratio
BMI	Body mass index
BP	Blood pressure
CI	Confidence intervals
DBP	Diastolic blood pressure
DBQ	Dietary Behavior Questionnaire
DXA	Dual-energy X-ray absorptiometry
FM	Fat mass
HDL-C	High-density lipoprotein cholesterol
HOMA-IR	Homeostatic model assessment for insulin resistance
IPAQ-SF	International Physical Activity Questionnaire
IQR	Interquartile range
LDL-C	Low-density lipoprotein cholesterol
LM	Lean mass
MET	Metabolic equivalent of task
MetS	Metabolic syndrome
NC	Neck circumference
ODI3	3% oxygen desaturation index
OSA	Obstructive sleep apnea
PA	Physical activity
PSQI	Pittsburgh Sleep Quality Index
SBP	Systolic blood pressure
SpO_2_	Peripheral oxygen saturation
STROBE	Strengthening the Reporting of Observational Studies in Epidemiology
TBF	Total body fat
VAT	Visceral adipose tissue
WC	Waist circumference
