# Supplementary material for: Associations of Dietary Patterns and Physical Activity with Sleep Quality and Metabolic Health Markers in Patients with Obstructive Sleep Apnea: An Exploratory Pilot Study
Source: Nutrients. 2026 Jan 26;18(3):409. doi: 10.3390/nu18030409 (PMC12899401; doi:10.3390/nu18030409)
Supplement: Supplementary file 1 [file nutrients-18-00409-s001.zip › nutrients-4071952-supplementary.pdf]

nutrients

MDPI

Article

Associations of Dietary Patterns and Physical Activity with Sleep Quality and Metabolic Health Markers in Patients with Obstructive Sleep Apnea

Li-Ang Lee, Yi-Ping Chao, Ruei-Shan Hu, Wan-Ni Lin, Hsueh-Yu Li, Li-Pang Chuang and Hai-Hua Chuang

Supplementary Table S1. Dietary Behavior Questionnaire.[28,29,81]
6

No.	Item	Short-Item	Response Options			
			Rarely	Occasion-ally	Often	Always
1	I eat three meals regularly.	Regular meals (3/day)	□	□	□	□
2	I avoid eating sweets or snacks.	Avoid sweets/snacks	□	□	□	□
3	I chew slowly when eating, at least 20 times per bite before swallowing.	Slow chewing (>20x)	□	□	□	□
4	When thirsty or hot, I drink plain water instead of soda, cola, sports drinks, or sweetened beverages.	Water intake (No sugary drinks)	□	□	□	□
5	I avoid fried or oily foods (e.g., cashews, peanuts, sunflower seeds, potato chips).	Avoid fried/oily food	□	□	□	□
6	I eat fruit every day.	Daily fruit intake	□	□	□	□
7	I eat green vegetables every day.	Daily vegetable intake	□	□	□	□
8	I eat late-night snacks.	Late-night eating	□	□	□	□
9	I eat while watching TV or reading magazines.	Distracted eating (TV)	□	□	□	□
10	I eat to relieve stress when I'm in a bad mood.	Emotional eating (stress)	□	□	□	□
11	I use food to reward myself or celebrate.	Reward eating	□	□	□	□
12	I shop for food only when I'm very hungry.	Shopping while hungry	□	□	□	□

Note: Participants were instructed to respond based on their eating habits over the past week, using a four-point frequency scale: "Always" (8 or more times out of 10), "Often" (6 to 8 times), "Occasionally" (3 to 5 times), and "Rarely" (1 to 2 times or never). Each item on the Dietary Behavior Questionnaire (DBQ) was scored from 0 to 3, corresponding to Rarely = 0, Occasionally = 1, Often = 2, and Always = 3. The total score, ranging from 0 to 36, was used to classify dietary behavior into four categories: Poor (0–12), Fair (13–20), Good (21–30), and Excellent (31–36).

References:

28. Health Promotion Administration. Conquering Obesity—Weight Loss Guidance Manual; Ministry of Health and Welfare, Taiwan: Taipei, Taiwan, 1997.

29. Huang, L.L.; Chen, Y.L.; Huang, C.T.; Yeh, D.F.; Lin, C.H. Analysis of the relationship between dietary habits and body mass index among continuing education students at a university of technology in central Taiwan. In Proceedings of 2018 Joint Annual Meeting and Academic Conference, Taichung, Taiwan, 14–19 October 2018; pp. 210-222.

81. Lin, C.C.; Ho, C.J. The Knowledge-Behavior Gap between Health Literacy and Diet: An Exploratory Study on Highly Educated Female Youth. J. Health Manag. 2018, 16, 15-36. https://www.airitilibrary.com/Article/Detail/18111947-201812-201903140007-201903140007-15-36

Disclaimer/Publisher's Note: The statements, opinions and data contained in all publications are solely those of the individual author(s) and contributor(s) and not of MDPI and/or the editor(s). MDPI and/or the editor(s) disclaim responsibility for any injury to people or property resulting from any ideas, methods, instructions or products referred to in the content.
